# Modeling and Optimization of β-Galactosidase Entrapping in Polydimethylsiloxane-Modified Silica Composites

**DOI:** 10.3390/ijms23105395

**Published:** 2022-05-12

**Authors:** Leszek Kadziński, Robert Łyżeń, Katarzyna Bury, Bogdan Banecki

**Affiliations:** Intercollegiate Faculty of Biotechnology of UG and MUG, University of Gdańsk, Abrahama Str. 58, 80-307 Gdansk, Poland; robert.lyzen@ug.edu.pl (R.Ł.); katarzyna.bury@ug.edu.pl (K.B.)

**Keywords:** silica composite, polydimethylsiloxane, beta-galactosidase, response surface methodology, lactose hydrolysis

## Abstract

Protein entrapment has multiple applications in enzymatic hydrolysis, drug delivery, etc. Here, we report the studies that successfully utilized the Box–Behnken design to model and optimize the parameters of β-galactosidase entrapment in sol–gel-derived silica composites. We have also demonstrated the influence of polymer–polydimethylsiloxane as a composite modifying agent on the activity of entrapped enzymes. We have determined how different sol-gel process parameters influence the activity of entrapped enzymes. The highest impact on β-galactosidase activity was exerted by the water:tetramethoxysilane ratio, followed by polydimethylsiloxane content. Optimized synthesis parameters have been utilized to obtain a composite with maximum β-galactosidase activity. Performed porosity studies have shown that the addition of polydimethylsiloxane increased the pore diameter. Microscopy studies demonstrated that polydimethylsiloxane-modified composites are softer and less rough. Studies of β-galactosidase activity using the o-NPG test showed statistically significant shifts in the enzyme temperature and pH profiles compared to the soluble form. An improvement in the reusability of the enzyme and a significant increase in the thermal stability was also observed. When lactose was used, a strong correlation was observed between the substrate concentration and the type of the catalyzed reaction. Moreover, we have demonstrated that the yields and rates of both lactose hydrolysis and galactooligosaccharides formation were correlated with reaction temperature and with the presence of polydimethylsiloxane. All these findings provide the opportunity for industrial use of optimized PDMS-modified silica composites in lactose elimination from dairy products, e.g., milk or whey.

## 1. Introduction

β-galactosidase (EC 3.2.1.23) is an enzyme of great importance for living organisms and the industry. The primary function of this protein in living cells is to hydrolyze lactose to glucose and galactose [1]. Industrially, this property is used to remove lactose from food products which is especially important for lactose-intolerant individuals. The ability of β-galactosidase to synthesize galactooligosaccharides (GOS) through a transgalactosylation reaction is also used [2,3]. As a result, the galactosyl moiety of lactose is transferred to a nucleophilic acceptor in order to produce a mixture of oligosaccharides with varying degrees of polymerization. The yield of galactooligosaccharides results from competition, as both reactions are catalyzed by β-galactosidase. It should be noted that the hydrolysis reaction is thermodynamically favored. Therefore, in order to obtain more GOS, it is necessary to carry out the reaction under optimal conditions for this process—for example, with a relatively high concentration of lactose [4].

β-galactosidase is a widely distributed enzyme. It is produced in animal, plant, fungal and bacterial cells. However, industrial applications mainly use enzymes produced by microorganisms. This is due to the ease of obtaining them while maintaining low costs and high efficiency of this process [5]. It is noteworthy that β-galactosidase from varying species of fungi and bacteria differs in terms of structure and properties. The enzyme derived from the fungus *Aspergillus oryzae* shows maximum activity at a temperature of about 55 °C and apH of 5.0. However, the analogous protein from the yeast *Kluyveromyces lactis* displays maximum activity at 30–35 °C and a pH of 6.5–7.0, respectively [6]. For this reason, fungal β-galactosidases are used more often to remove lactose from whey products and yeast enzymes for dairy products.

In industry, enzymes are often added directly during the production process, meaning they are either inactivated or remain in the end product. However, this makes it impossible to use them again. Therefore, reusability is one of the most important parameters for the industrial application of enzymes. This approach requires the use of a carrier for the active protein to take part in the physical (e.g., entrapment, adsorption) or chemical (e.g., cross linking, covalent binding) immobilization of the enzyme.

Physical adsorption is based on the weak interaction between the support and the enzyme. Support is relatively cheap, but this method often requires its regeneration and reloading with protein (due to protein leakage). Covalent bonding is the irreversible enzyme immobilization technique. Matrices for such immobilization usually include agarose, cellulose, poly(vinyl chloride), ion exchange resins and porous glass. The functional group involved in the binding of the enzyme should not take part in catalytic activity. This technique provides enhanced stability and reusability of composite but requires specific knowledge to perform chemical modifications. On the other hand, entrapment is another irreversible method of enzyme immobilization. In this case, the enzyme is entrapped inside the support, but substrates and products can pass through it. In this method, the enzyme does not interact directly with the matrix, it allows an increase in stability, and composites show reusability and chemical modifications in support can create a protein-friendly microenvironment. This technique is relatively cheap and easy to use. The greatest disadvantage of this method is that there is a possibility of leakage of low molecular weight enzymes from the matrix [7]. The industry favors cost-effective and easy methods that maintain or improve catalytic activity and ensure reusability [8].

An example of such carriers is silica composites obtained by the sol–gel method. The primary advantages of using these materials are the ease of their synthesis. The sol–gel method usually runs at room temperature and allows the use of aqueous emulsions of precursors and buffered solutions of entrapped enzymes [9,10,11]. First, the hydrolysis of the precursors is carried out, which leads to the formation of a colloidal suspension of the compounds used, called the sol. At this stage, it is possible to add the enzyme solution followed by polycondensation and the consequent formation of a gel [12]. The gel undergoes the removal of excess of water and alcohol formed in the earlier stages via evaporation, which leads to the formation of a xerogel (evaporation under normal conditions) or an aerogel (evaporation under supercritical conditions) [13,14]. A very important parameter in the synthesis of silica composites is the possibility of using various modifiers (organic or inorganic). In this work, the influence of polydimethylsiloxane on the structure and properties of the xerogel itself, as well as on the activity and stability of the entrapped enzyme (β-galactosidase), was investigated.

**Figure 1:**
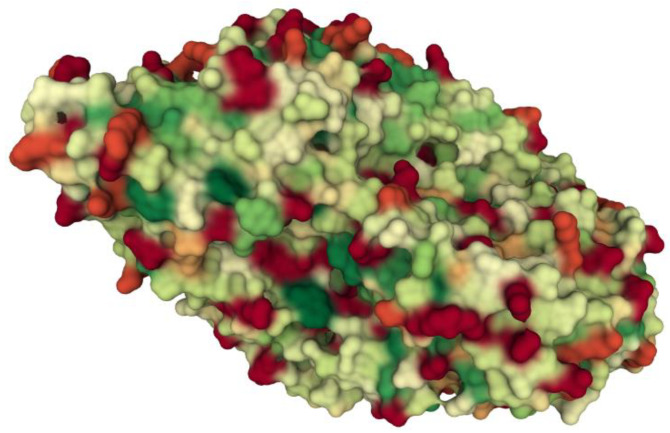
Hydrophobicity of β-galactosidase from *Aspergillus oryzae* (PDB ID: 4IUG; [21]. Molecular surface representation (red—hydrophilic residues; green—hydrophobic residues).

Hydroxylated polydimethylsiloxane forms (PDMS) of various chain lengths have been used to modify silica composites over the years. PDMS has been shown to influence the process of drug release from xerogels [15,16,17,18]. Using this polymer as a modifier resulted mainly from the fact that it is considered to be non-toxic, biocompatible and hydrophobic. However, in our previous studies, we have shown that PDMS can interact with proteins (e.g., collagen). The interactions are inversely proportional to the length of the chain. They are strong enough to capture collagen from the solution, but weak enough to not cause changes in the structure and functions of collagen. This suggests that these interactions are hydrophobic in nature [19]. β-galactosidase from *Aspergillus oryzae* is not a very hydrophobic protein, but on the surface it has regions that can interact with PDMS methyl residues, which may increase the stability of the entrapped enzyme (Figure 1) [20]. There are no reports in the literature on the influence of polydimethylsiloxane on the activity and stability of proteins enclosed in a silica composite, which this work tries to complete.

One of the key problems in working with silica composites is the development of an optimal synthesis procedure. This is a time-consuming and resource-demanding process because of the enormous number of possible variants. These variants may consider parameters such as the content of the precursor, the molar ratio of the precursor and water, the presence and content of modifiers, the type and concentration of catalysts, as well as the drying time and temperature of the material formed by the sol–gel method. A useful methodology is the design of experiments (DoE) which analyzes the impact of individual variables on the course of the process. It also indicates the interactions between the parameters, which is not possible in the one-factor analysis. Examples of such designs are the Box–Behnken design (BBD) and central composite design (CCD). The traditional univariate procedure is not satisfactory as it only changes the level in one factor while the others remain unchanged. However, the full factorial design involves many experiments. George E. P. Box and Donald Behnken reconciled these issues when in 1960 proposed a three-factorial analysis [22,23]. The major advantage of their analysis was the reduction in the number of experiments. The BBD does not contain combinations in which all the variables are at extreme levels (either the lowest or the highest) [24,25].

In this work, we showed the Box–Behnken design application in the synthesis of silica composites containing the enzyme β-galactosidase. This methodology was also used to optimize the composite synthesis process in terms of the activity of entrapped protein.

## 2. Results

### 2.1. Optimization of β-Galactosidase Entrapment in Sol–Gel-Derived Silica Composites

#### 2.1.1. Modeling of the Influence of Synthesis Parameters on Silica Composite Gelation Time

**Figure 2:**
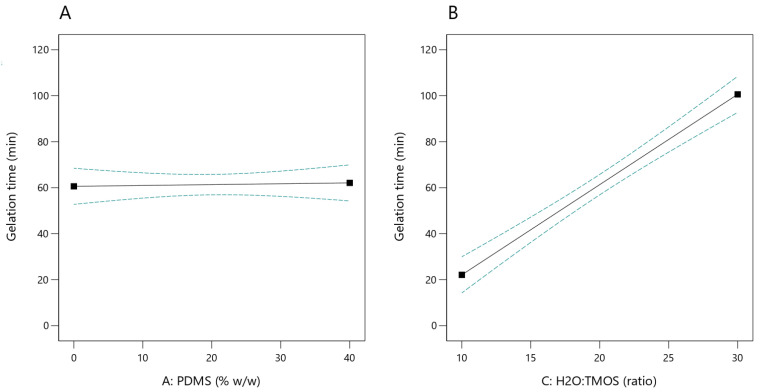
Effect of PDMS content (**A**) and H_2_O:TMOS molar ratio (**B**) on the sol gelation time (the dashed lines indicate the 95% confidence interval).

Under the experimental conditions tested, the gelation time was in the range of 10–112 min. The statistical analysis of the model showed that it is significant (*p*-value < 0.0001) with non-significant lack of fit. A statistically significant relationship was demonstrated between the molar ratio of water to TMOS and the gelation time (*p*-value < 0.0001; Figure 2B)—the higher the ratio, the longer the sol-to-gel transition time. It was shown that the content of PDMS had no significant impact on this process (*p*-value = 0.8064; Figure 2A).

#### 2.1.2. Modelling the Influence of Synthesis Parameters on Entrapped β-Galactosidase Activity

Synthesis of silica composites with different process parameters of PDMS content, drying time and H_2_O:TMOS molar ratio gave β-galactosidase activity from 2.13 to 22.16 ALU/g (Table 1).

**Figure 3:**
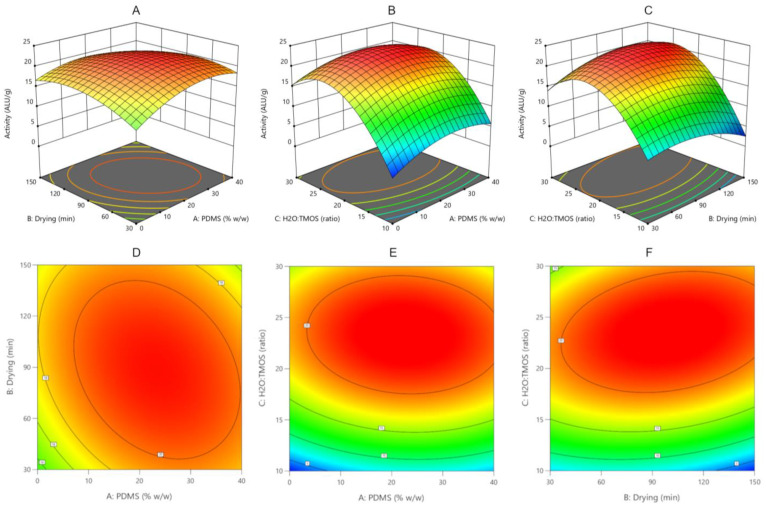
Response surface plots (**A**–**C**) and corresponding contour plots (**D**–**F**) shows the combined effect of PDMS content and drying time (**A**,**D**), PDMS content and H_2_O:TMOS ratio (**B**,**E**) and drying time and H_2_O:TMOS ratio (**C**,**F**). Other variables are fixed at their respective center point (PDMS: 20% *w*/*w*; drying time: 90 min; H_2_O:TMOS ratio: 20).

Statistical analysis of the Box–Behnken design model showed that the model is significant. The input variables studied have a statistically significant effect on the enzyme activity (except drying time in the analyzed range). The highest impact on β-galactosidase activity was exerted by H_2_O:TMOS molar ratio followed by PDMS content. Analysis of the response surface graphs (Figure 3) and the fitted model equation indicates that the maximum activity should be determined when PDMS content is in the middle of the tested range (20–30% *w*/*w*). Higher H_2_O:TMOS molar ratio increases the enzyme activity but only until reaching the value of 23.3 ALU/g. A further increase in the ratio value causes a decrease in enzyme activity.

It was found that quadratic relations are significant and a statistically significant interaction occurs between all tested variables (Table 2). The lack of fit of the model is insignificant (F-value of 2.37 implies the lack of fit is not significant relative to the pure error). Values of the adjusted and predicted coefficient of determination R2 are high and in good agreement (0.9949 and 0.9759, respectively). The model has a high precision of 52.13, indicating an adequate signal, meaning it can be used to predict the response. Equation of the model:

β-galactosidase activity (ALU/g) = 21.85 + 1.03 × A + 0.1294 × B + 5.87 × C − 1.56 × AB − 0.6302 × AC + 2.11 × BC − 3.14 × A² − 2.74 × B² − 8.71 × C²(1)

where A—PDMS content (wt%); B—drying time (min); and C—H_2_O:TMOS (molar ratio).

#### 2.1.3. Model Optimization

The mathematical models recovered in the previous steps were used to optimize the synthesis parameters. The major goal was to maximize the enzyme activity and the minor goal was to achieve a gelation time in the range of 60–90 min. Numerical optimization was performed resulting in good desirability of fitting (0.982). Observed enzyme activities were in good agreement with predicted ones, both for the composite with PDMS (X1) and without PDMS (X2). Optimized synthesis parameters, as well as predicted and determined values of responses, are presented in Table 3.

### 2.2. Physicochemical Properties of Optimized Silica Composites

#### 2.2.1. Surface Area and Pores Size Distribution Determination

**Figure 4:**
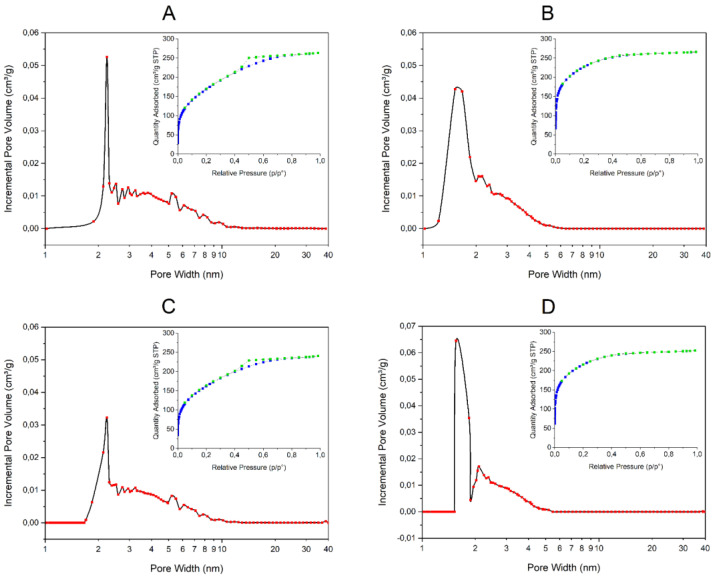
Pore size distribution curves and nitrogen adsorption−desorption isotherms (inserts) of silica composites: (**A**) X1 (with both β-galactosidase and PDMS); (**B**) X2 (with β-galactosidase and without PDMS); (**C**) X3 (without β-galactosidase and with PDMS); (**D**) X4 (without both β-galactosidase and PDMS).

Nitrogen adsorption–desorption isotherms for the different obtained composites are presented in Figure 4. According to the Brunauer, Deming, Deming and Teller (BDDT) classification, the isotherms for composites X2 (with β-galactosidase and without PDMS) and X4 (without both β-galactosidase and PDMS) are of type I (inserts on Figure 4B,D, respectively). For this type of isotherm, it is characteristic that a high volume of nitrogen is adsorbed at low relative pressure. It indicates that analyzed materials are microporous (pore radii < 2 nm), which has been confirmed by porosity distribution by the density functional theory (DFT) method (Figure 4B,D, respectively). Moreover, the lack of hysteresis in the desorption curve suggests the pores are as large as the openings leading into them. Determined BET surface areas for composites X2 and X4 are 781.5 m²/g and 746.4 m²/g, respectively.

Composites X1 (with both β-galactosidase and PDMS) and X3 (without β-galactosidase and with PDMS) present adsorption–desorption isotherms more similar to type IV, which are characteristic for mesoporous materials with pore radii > 2 nm (inserts on Figure 4A,C, respectively). It is in good agreement with porosity distribution by the DFT method (Figure 4A,C, respectively). The existence of hysteresis in the desorption curve indicates that pore cavities are larger in diameter than their openings. Determined BET surface areas for composites X1 and X3 are 617.5 m²/g and 587.5 m²/g, respectively.

#### 2.2.2. Atomic Force Microscopy

**Figure 5:**
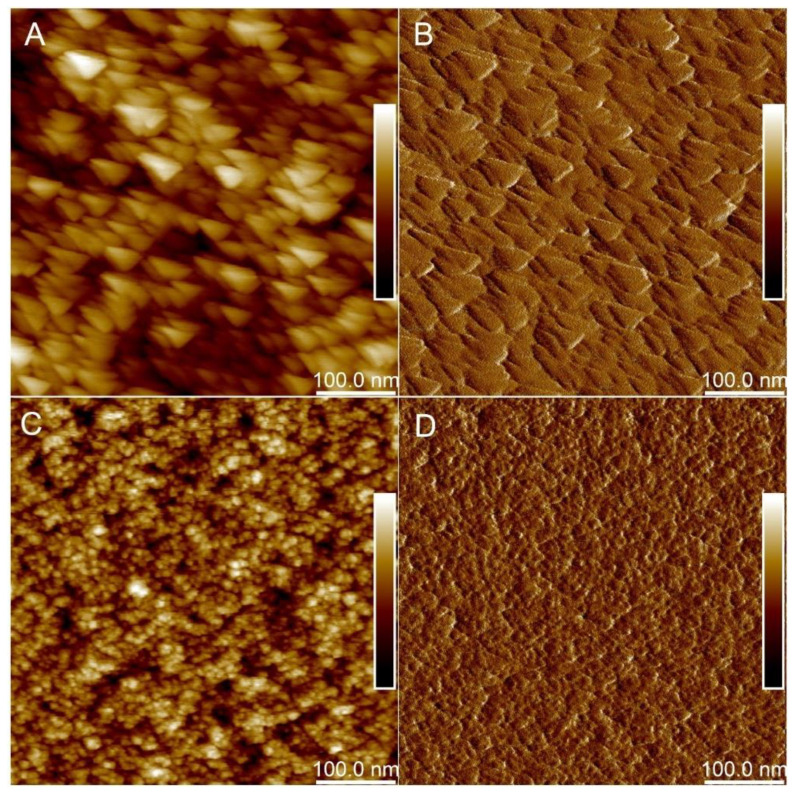
AFM micrographs of silica composites: (**A**) X1 in HS mode (with both β-galactosidase and PDMS); (**B**) X1 in PFE mode; (**C**) X2 in HS mode (with β-galactosidase and without PDMS); (**D**) X2 in PFE mode (with β-galactosidase and without PDMS). Dark colors indicate depressions and light color protrusions.

The AFM Height Sensor images (Figure 5A,C) and corresponding Peak Force Error images (Figure 5B,D) reveal that the addition of PDMS leads to a more flat surface of the composite. The value of mean roughness (Ra) obtained for the composite modified with 22.9% (*w*/*w*) PDMS with entrapped β-galactosidase (X1) was estimated at 1.009 nm, but for the composite without PDMS (X2)—1.619 nm. Calculated surface stiffness was 0.009 and 0.012 N/m for composites X1 and X2, respectively. It is in good agreement with Young’s moduli determinations. Composite X1 through the addition of PDMS is softer than composite X2, with Young’s modulus in the ranges of 342.1–453.8 kPa. Composite X2, which consisted of much stiffer silica, has Young’s modulus between 383.913–589.385 kPa.

### 2.3. Enzymatic Activity of β-Galactosidase in Silica Composites

#### 2.3.1. Properties of Entrapped β-Galactosidase in PDMS-Modified and Non-Modified Silica Composites

**Figure 6:**
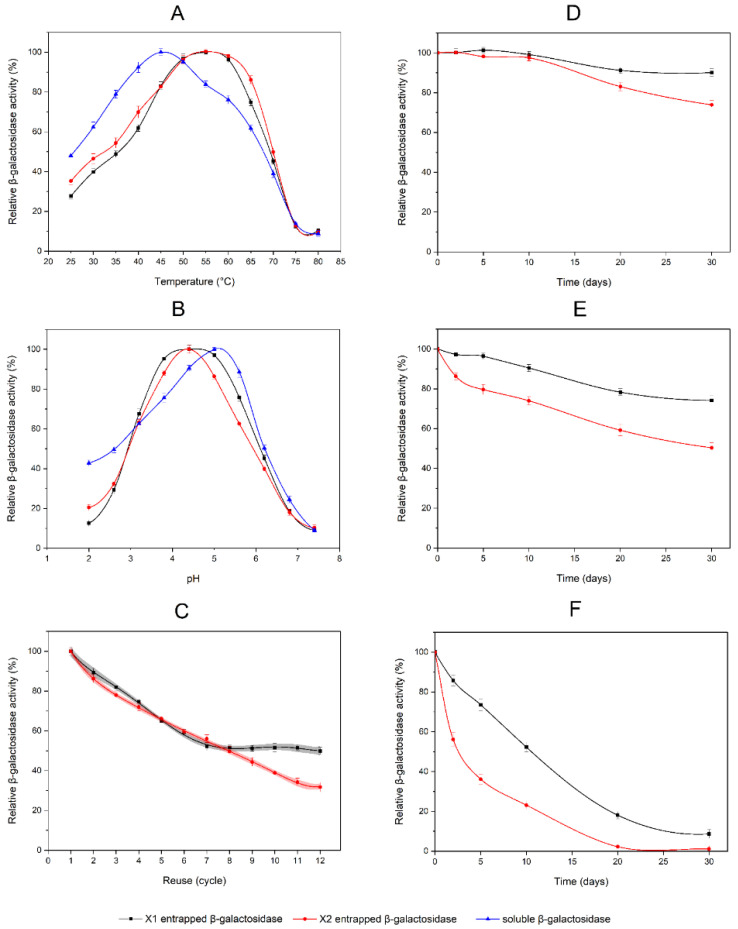
Properties of entrapped β-galactosidase in PDMS-modified and non-modified silica composites: (**A**) temperature profiles; (**B**) pH profiles; (**C**) reusability of entrapped β-galactosidase—the 95% confidence bands are marked in gray and light red; (**D**) β-galactosidase stability tests at 4 °C; (**E**) β-galactosidase stability tests at 25 °C; (**F**) β-galactosidase stability tests at 40 °C. The data shown are the means of three independent experiments ± standard deviations. Black squares (▬■▬) represent data for β-galactosidase entrapped in the PDMS-modified composite—X1; red circles (▬●▬) represent data for β-galactosidase entrapped in the non-modified composite—X2; blue triangles (▬▲▬) represent data for free soluble β-galactosidase.

The hydrolyses of o-NPG by entrapped β-galactosidase in PDMS-modified and non-modified composites, as well as by soluble β-galactosidase, were determined as a function of temperature and pH. The influence of temperature was studied in a range of 25 to 80 °C. Temperature profiles for entrapped enzymes were broader for relatively high temperatures of 50–60 °C than for soluble enzymes—40–50 °C. It resulted in a shift of optimal hydrolysis temperature from 45 °C to 55 °C for soluble and entrapped β-galactosidase, respectively (Figure 6A). These findings indicate that the entrapped enzymes possessed better heat tolerance than the soluble form. However, the enzyme activities determined in PDMS-modified composites were 11.1% higher than in non-modified ones (44.85 and 40.38 ALU/g, respectively). The effect of pH on the activity was determined between 2.0 to 7.4 for entrapped and soluble forms of β-galactosidase. There was an observed shift of optimal pH to the acidic region for entrapped enzymes relative to soluble form (maximum activity at pH 4.4 and 5.0, respectively). Furthermore, the enzyme activity profile against pH had a distinct peak at a maximum for the PDMS-free silica composite. The PDMS-modified composite had a pH profile with a flattened top which indicates a broader range of optimal pH (Figure 6B).

PDMS-modified composites (X1) containing β-galactosidase and silica composites (X2) with entrapped β-galactosidase were used for 12 consecutive batches of hydrolytic reactions with o-NPG. The relative activities of entrapped β-galactosidases, on reuse, are shown in Figure 6C, and the activity of the first batch was taken as 100%. For the first six cycles, there was no significant difference in the rates of the loss of enzyme activity between composites X1 and X2. The relative activity of β-galactosidase entrapped in PDMS-modified composites was decreased to 59.2% (±1.7). The determined relative activity of the enzyme in the non-modified composite was 59.5% (±1.3). A further decrease in activity was observed in cycles 7–12 for composite X2. However, for composite X1, the decrease was inhibited and the relative activity of β-galactosidase stabilized at the level of 51.4% (±0.8).

Several 30-day thermal inactivation studies were performed at three temperature points (4 °C, 25 °C and 40 °C) for composites X1 and X2. During incubation at 4 °C, the PDMS-modified composite (X1) remained at 90.1% (±1.9) of β-galactosidase activity. Enzymes in the non-modified composite (X2) were stable for 10 days. Subsequently, a slow rate of activity decrease was observed, which resulted in the relative activity of 73.9% (±2.2) after 30 days (Figure 6D). Studies at 25 °C demonstrated that after 30 days of incubation, the relative activity of the enzyme in composite X2 was decreased to 50.4% (±2.4), while β-galactosidase in X1 composite remained 74.2% (±2.3) of its initial activity (Figure 6E). At 40 °C, β-galactosidase in both the PDMS-modified (X1) and non-modified composite (X2) was gradually deactivated. After 20 days, there was no detectable activity of β-galactosidase in composite X2, while the residual activity in composite X1 was 18.1% (±1.8). Even after 30 days at 40 °C, the trace activity of the entrapped enzyme was 8.6% (±2.2) for the PDMS-modified composite (Figure 6F).

#### 2.3.2. Influence of PDMS as a Silica Composite Modifier on the Activity of β-Galactosidase by Determination of Lactose Hydrolysis Products

**Figure 7:**
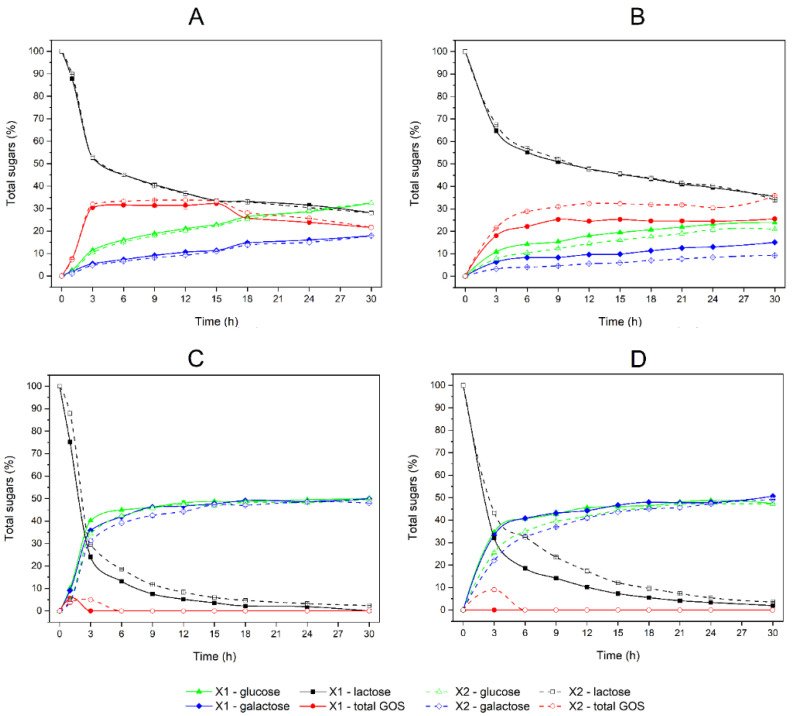
Reaction kinetics of lactose hydrolysis and GOS formation catalyzed by β-galactosidase entrapped in the PDMS-modified composite X1 (solid lines) and non-modified composite X2 (dashed lines): (**A**) hydrolysis of 250 g/L lactose at pH 4.5 and 55 °C; (**B**) hydrolysis of 250 g/L lactose at pH 4.5 and 30 °C; (**C**) hydrolysis of 50 g/L lactose at pH 4.5 and 55 °C; (**D**) hydrolysis of 50 g/L lactose at pH 4.5 and 30 °C. The data shown are the means of two independent experiments ± standard deviations. Black squares represent data for lactose content (%); green triangles represent data for glucose content (%); blue diamonds represent data for galactose content (%); and red circles represent data for total galactooligosaccharides (GOS) content (%).

The hydrolysis of lactose by entrapped β-galactosidase in PDMS-modified and non-modified composites was determined by HPLC. This technique allowed for the simultaneous detection and quantification of the products of the transgalactosylation reaction—galactooligosaccharides (GOS). About 80% of detected GOS was trisaccharides (3-GOS), about 10% was tetrasaccharides (4-GOS), the rest were higher oligosaccharides. In the case of the reaction carried out with the use of the substrate (lactose) at a concentration of 250 g/L and 55 °C, no statistically significant difference was observed in the kinetics of lactose loss between the composites containing PDMS (X1) and the unmodified composites (X2). However, it was noticed that the modification of the composite with PDMS shifted the equilibrium of the reaction towards the hydrolysis of lactose. This resulted in a several percent increase in glucose and galactose content (e.g., 5.4% and 12.6%, respectively, after 9 h of incubation) and a corresponding decrease in GOS content (−7.0% after 9 h of incubation) in the reaction mixture, compared to the non-modified composite (Figure 7A). This relationship was even more evident when the reaction was carried out at 30 °C. When the PDMS-modified composite (X1) was used as an enzyme carrier, there was an observed 25.2% increase in glucose and 82.2% increase in galactose content after 9 h of incubation, relative to the non-modified composite (X2). On the other hand, the content of GOS after 9 h was decreased by 18.3% while the kinetic of lactose content did not change significantly (Figure 7B).

When a 50 g/L concentration of lactose was used, the production of GOS was strongly decreased and the equilibrium of the reaction shifted towards the hydrolysis of lactose. GOS was only formed in the initial phase of incubation, when the substrate concentration was still relatively high. At the temperature of 55 °C, GOS was synthesized by the enzyme from both the X1 and X2 composites, while at the temperature of 30 °C only the β-galactosidase enclosed in the X2 composite was able to synthesize GOS. In all cases, the GOS was unstable and after 6 h of incubation, it was not observed in the incubation mixtures. Under these conditions, the lactose hydrolysis reaction was thermodynamically favored. The kinetics of this process showed a strong statistical dependence on the presence or absence of PDMS as a composite modifier. When the PDMS-modified composite X1 was used as an enzyme carrier, there was a statistically significant increase in glucose and galactose content after the first 6 h of incubation (*p* < 0.05 at 55 °C and *p* < 0.01 at 30 °C), relative to the non-modified composite X2. On the other hand, the rate of lactose hydrolysis was significantly higher (*p* < 0.005 at 55 °C and *p* < 0.001 at 30 °C) for enzymes entrapped in the X1 composite, relative to X2 (Figure 7C,D).

## 3. Discussion

The Box–Behnken design has been successfully utilized to model and optimize the parameters of β-galactosidase entrapment in sol–gel-derived silica composites. In the first step, the effect of the H_2_O:TMOS ratio factor and PDMS content on the gelation time of the composites was examined. It was demonstrated that in the analyzed range, the PDMS content did not significantly affect the gelling process; however, the process was strongly correlated with the H_2_O:TMOS ratio (the higher the value of this factor, the longer the gelation time). Increased values of this ratio reduce hydrolysis and condensation rates; therefore, there is an increase in the gelation time. This is in good agreement with data from other gelation studies [26]. Next, it was determined how different process parameters of PDMS content, drying time and H_2_O:TMOS molar ratio influence the activity of entrapped β-galactosidase. The highest impact on β-galactosidase activity was exerted by H_2_O:TMOS molar ratio followed by PDMS content. It was also observed that statistically significant interaction occurs between all the tested variables. This research approach has been used in the past [27], but the entrapping process of β-galactosidase has not been modeled or optimized so far, regarding PDMS modification of a composite. Optimized synthesis parameters have been utilized to produce composites with maximum β-galactosidase activity with a desired gelation time and a series of control composites (without PDMS, without β-galactosidase or both). The obtained composites were then subjected to physicochemical tests to examine their structure and surface.

Although H_2_O:TMOS molar ratio influences the activity of entrapped β-galactosidase. It also affects the entrapment process itself. The structure of pore size and mechanical strength of SiO_2_ composite are critically controlled by the molar ratio of H_2_O:TMOS during the sol–gel synthesis. The adequate molar ratio of H_2_O:TMOS is also required to complete the polymerization of silanes. PDMS in the oxide network also influences the microstructure, stability and bioactivity of the composite matrix. It provides more hydrophobic microenvironment of carrier, which generally stabilizes enzyme. There are numerous parameters of the sol–gel process such as temperature, ionic strength, solvent used, aging time, etc. which can affect the entrapment [28], but in the presented study they were fixed to maintain the activity of immobilized protein.

Porosity studies have shown that composites without PDMS are microporous with pore radii < 2 nm. The addition of PDMS as a composite modifier resulted in an increase in the pore diameter, regardless of the presence of an entrapped enzyme. This agrees with other published data where a relationship was observed between PDMS content and pore diameter increase [12,15].

Microscopy studies have shown that PDMS-modified composites are softer (with lower average Young’s moduli) and less rough than non-modified silica composites.

Studies of β-galactosidase activity using the o-NPG test showed statistically significant shifts in the entrapped enzyme activity profiles as a function of temperature and pH compared to the soluble form. Such shifts have been reported in other published works. Depending on the origin of the enzyme and the method of immobilization, the direction of this shift (especially in the case of pH) may vary. For example, Song et al. have observed a shift to the basic region after the immobilization of β-galactosidase [29]. However, there were also reports of shifts to the acidic region [30]. These changes in the optimum pH may have depended on the charge of the enzyme and the solid support. The silica surface at pH 5 is weakly negatively charged and PDMS as a modifier is also a negatively charged compound. So, PDMS-modified composites, being more negatively charged than the non-modified silica, can attract the H^+^ more efficiently in the reactive solution to its nearby surroundings. This may lead to a decrease in the pH of the area on which the enzyme is entrapped. This explains the widening of the pH range for PDMS-modified composites in which the enzyme activity is the highest. For composites containing PDMS, there was also an observed improvement in the reusability of the enzyme and a significant increase in the thermal stability of entrapped β-galactosidase. These findings allow for the industrial use of the optimized PDMS-modified silica composite.

When lactose was used as the substrate for the β-galactosidase entrapped in the composite, a strong correlation was noted between the substrate concentration and the main enzyme catalyzed reaction. When the lactose concentration was high (250 g/L in our research), the reaction equilibrium shifted towards the transgalactosylation reaction. This resulted in the formation of galacto-oligosaccharides up to the level of about 30% of all sugars. This is in agreement with other published data [31,32]; however, in our studies, we have demonstrated that the yield and rate of GOS formation were correlated to reaction temperature (which was not shown by Bolognesi et al. [32]) and with the addition of PDMS as a silica composite modifier. When the entrapped enzyme catalyzed lactose at a low concentration (50 g/L), the primary reaction was hydrolysis of substrate to glucose and galactose. Only small amounts of GOS were synthesized at the initial phase of incubation. There was also a statistically significant influence of PDMS on the reaction rate observed.

## 4. Materials and Methods

### 4.1. Materials

Tolerase^®^ L, a solid preparation of β-galactosidase from *Aspergillus oryzae* (specific activity: 130,000 ALU/g) was purchased from DSM Ltd. (Herleen, The Netherlands). Tetramethoxysilane (TMOS, 98%), polydimethylsiloxane–hydroxy terminated (PDMS, viscosity ~25 cSt), sodium acetate (≥99%), methanol gradient grade for HPLC (≥99%), acetonitrile gradient grade suitable for HPLC (≥99.9%), o-nitrophenyl-d-galactopyranoside (99%, o-NPG), o-nitrophenol (99%, o-NP), D-(+)-Glucose (≥99.5%), D-(+)-Galactose (≥98%) and D-Lactose monohydrate (≥98.0%) were purchased from Sigma-Aldrich (Poznań, Poland).

### 4.2. Optimization of β-Galactosidase Entrapment in Sol–Gel-Derived Silica Composites

#### 4.2.1. Silica Composites Preparation

Enzyme entrapped silica composites were synthesized using the one-step sol–gel process. Briefly, the silica precursor (TMOS) was combined with ddH_2_O at desired H_2_O:TMOS molar ratios. These mixtures were acidified with the addition of about 100 µL of 0.1 N HCl per 15 mL of final sol volume. The resulting solutions were first mixed on a magnetic stirrer for 10 min at room temperature, then they were sonicated for 10 min at 30 °C. Next, methanol was added to the final concentration of 10% (*w*/*v*) and 25 cSt PDMS to the appropriate wt%. Obtained solutions were sonicated for another 10 min at 30 °C and left to cool down to room temperature. These sols were then combined with solutions of β-galactosidase in sodium acetate buffer (100 mM, pH 4.5; final enzyme content in composites—1 mg/g) and poured into Petri dishes. The final volumes of each obtained sol were 15 mL. The addition of acetate buffer promotes sol gelation, after which gels were placed at a temperature of 4 °C for overnight aging. Next, gels were placed at room temperature and they were dried for the appropriate time (specific concentrations of PDMS, H_2_O:TMOS molar ratios and drying times are presented in Table 4).

#### 4.2.2. Modeling of β-Galactosidase Entrapping in PDMS-Modified Silica Composites

The analysis of the effects of PDMS content, gel drying time and H_2_O:TMOS molar ratio on the enzyme activity was performed using a Box–Behnken design (BBD) with response surface methodology (RSM). It is a set of methods that allows one to minimize the number of necessary experimental runs and to optimize processes more efficiently in terms of costs and labor by fitting the mathematical model and determining a regression equation containing linear and quadratic effects and interactions between variables. The graphic representation of such a regression equation is called response surface [33,34]. Levels of independent variables are shown in Table 4 and the experimental matrix is shown in Table 1. The Box–Behnken design is rotatable, but it contains regions of poor prediction quality. Its “missing corners” may be useful for determining when the experimenter should avoid combined factor extremes. Therefore, the levels of all 3 variables were selected to exclude extreme values. PDMS content was tested up to 40 wt% (level 1) based on published data [15,16]. On the other hand, no PDMS addition was defined as level −1. The H_2_O:TMOS ratio was tested in the range of 10 to 30 and the drying time in the range of 30 to 150 min, based on preliminary tests taking into account the gelation time and the degree of drying of the composite. In the BBD, the third level (level 0) is always the average value for the analyzed range. The relationship between the preparation parameters (independent variables) and β-galactosidase activity (the response) was fitted to a predictive second order polynomial equation:

(2) $$Y_{i}=\beta_{0}+\overset{n}{\underset{i=1}{\sum }}\beta_{i}X_{i}+\overset{n}{\underset{i=1}{\sum }}\beta_{ii}X_{i}^{2}+\overset{n-1}{\underset{i=1}{\sum }}\overset{n}{\underset{j=i+1}{\sum }}\beta_{ij}X_{i}X_{j}$$

where *Y_i_* is the predicted response (β-galactosidase activity); subscripts *i* and *j* have values from 1 to the number of variables; *β*_0_ is a constant; *β_i_* is the linear coefficient; *β_ii_* is the quadratic coefficient; *β_ij_* is the cross-product coefficient; *n* is the number of factors; and *X_i_* and *X_j_* are the coded dimensionless values of the analyzed variables.

The software Design-Expert 13 (Stat-Ease, Minneapolis, MN, USA) was used for experimental design, the analysis of variance (ANOVA) and the graphical analysis of the data. The statistical significance of the model was expressed by an *F*-test and the quality of its fit was evaluated by the coefficient of determination *R*^2^. The significance of the regression coefficients was tested by the Student’s *t*-test and the *p* values were used to determine the significance of each coefficient.

#### 4.2.3. Optimization of β-Galactosidase Entrapping

To optimize the β-galactosidase entrapping process, numerical optimization was carried out using Design-Expert 13 software (Stat-Ease, USA). The major goal was to maximize the enzyme activity and the minor goal was to obtain a gelation time in the range of 60–90 min. Goals were combined into the overall desirability function. The program aims to maximize this feature. The target search starts at a random starting point and goes up the steepest slope to the maximum. As a result, the software determines such composite synthesis parameters that the assumed goals are met.

### 4.3. Determination of Physicochemical Properties of Optimized Enzyme-Containing Silica Composites

Based on the synthesis process parameters modeled and optimized as described in Section 4.2, a composite containing entrapped β-galactosidase (X1) was obtained. To investigate the effect of PDMS on the properties and activity of the material, a PDMS-free composite (X2) was also synthesized. Two control composites were also obtained: X3—without β-galactosidase; and X4—without β-galactosidase and PDMS. All of these material’s variants were analyzed in order to test their physicochemical properties.

#### 4.3.1. Surface Area and Pores Size Distribution Determination

Specific surface area and pore size distribution values were obtained from N_2_ adsorption–desorption isotherms at 77 K (Micromeritics Gemini VI Analyzer). The Brunauer–Deming–Deming–Teller (BDDT) methodology was used for the gas adsorption isotherm classification, the surface area was determined by the BET equation and the pore size distribution was determined by the BJH and DFT methods [35,36]. Before the measurement, the samples were dried at 70 °C in the flow of helium for 6 h. Immediately before the measurement, the samples were desorbed in the apparatus for 30 min at room temperature under vacuum conditions.

#### 4.3.2. Atomic Force Microscopy (AFM)

Composites obtained in the form of a film (according to the procedure described in Section 4.2.1 and with the optimized parameters obtained according to Section 4.2.3) were crushed to obtain fragments with an area of about 4 mm^2^. The obtained fragments were placed on freshly cleaved mica substrates and characterized by using atomic force microscopy. AFM of composites were performed using BioScope Resolve AFM (Bruker, Bremen, Germany) at 23 °C in air. The ScanAsyst-Air probe (Bruker) was used for atomic force imaging (resonant frequency f0 = 70 kHz; spring constant k = 0.4 N/m). Images were taken at 512 × 512 pixels with a PeakForce Tapping frequency of 1 kHz and an amplitude of 150 nm. A height sensor signal was used to display the protein image using NanoScope Analysis v1.9 (Bruker, Bremen, Germany).

### 4.4. Enzymatic Activity of β-Galactosidase in Silica Composites

#### 4.4.1. o-NPG Assay

The optimal pH values for both entrapped and free β-galactosidase were studied using o-NPG in the pH range of 2.0–7.4 in 0.1 M citrate-phosphate buffer. Under standard conditions, 0.1 M sodium acetate buffer with pH 4.5 was used and activity was observed. The optimal temperature was studied for both free and entrapped enzymes by assaying the enzyme activity with o-NPG at a temperature between 25 and 80 °C in their optimal pH buffers for 5 min. The temperature stability (thermal inactivation) was studied by incubating PDMS-modified and non-modified silica composites with entrapped enzymes at various temperatures (4, 25 and 40 °C) at different time intervals, followed by residual activity assay under standard conditions.

Briefly, about 10.0 mg of silica composite was weighed and washed twice with 1.0 mL of 0.1 M sodium acetate buffer (pH 4.5). Next, the washing buffer was replaced with 1.0 mL of o-NPG solution (37.0 mg of o-NPG dissolved in 0.2 mL of dimethyl sulfoxide and made up to 10 mL with 0.1 M sodium acetate buffer, pH 4.5) equilibrated to target reaction temperature (usually 37.0 °C) which started the reaction. Reaction tubes were incubated in thermomixer at target temperature (usually 37.0 °C) with mixing at 800 rpm. After an appropriate amount of time (5 or 10 min), 100 µL of the reaction mixture was transferred to 100 µL of 10% (*w*/*v*) Na_2_CO_3_ to stop the reaction. Next, the tube content was mixed and 800 µLddH_2_O was added. The contents of the tube were transferred to spectrophotometric cuvette and liberated 2-nitrophenol was measured at 420 nm, after which its concentration was calculated from a plot constructed using standard 2-nitrophenol solutions. One unit of enzyme activity (ALU) is defined as the amount of enzyme catalyzing the hydrolysis of 1 µM of o-NPG per minute under testing conditions.

For reusability assessment, the entrapped enzyme was repeatedly used 12 times and the residual activity was measured with o-NPG as the substrate. After each assay, the composite was properly washed with 0.1 M sodium acetate buffer, pH 4.5, to remove any attached substrate.

#### 4.4.2. Lactose Hydrolysis

The efficiency of lactose hydrolysis was studied using composites with entrapped β-galactosidase—X1 (modified with PDMS) and X2 (without PDMS). The experiment was performed with a substrate at different concentrations: 5% and 25% (*w*/*v*). In each variant, about 100.0 mg of silica composite was weighed and placed in a 2 mL Eppendorf tube. Next, 800 µL of lactose solution of an appropriate concentration was added and the tube was placed in a thermomixer. Incubation was carried out at a temperature of 55 °C and 30 °C with continuous mixing at 800 rpm. Aliquots were withdrawn at regular time intervals and released glucose, galactose and galactooligosaccharides as well as remaining lactose were estimated by HPLC with evaporative light scattering detector. For the substrate with initial lactose concentration of 5% (*w*/*v*), 20 µL of incubation solution was mixed with 180 µL of ddH_2_O. Following this, 10 µL aliquots of 25% (*w*/*v*) lactose were transferred to 390 µL of ddH_2_O.

### 4.5. HPLC Determination of Sugars

HPLC determinations were performed on the Supelcosil™ LC-NH2 HPLC Column —5 μm particle size, L × I.D. 25 cm × 4.6 mm from Supelco (Poznań, Poland) and Nexera XR High Performance Liquid Chromatograph coupled with ELSD detector from Shimadzu (Tokyo, Japan). Separations were performed with a mobile phase flow of 1.5 mL/min in gradient mode. The gradient program was as follows: 0–25 min—linear from 80% B to 50% B; 25–26 min—linear from 50% B to 80% B; 26–30 min—isocratic 80% B, where A—ddH_2_O and B—acetonitrile. ELSD was operating at 40 °C with air as a nebulizing gas (pressure: 3.5 bar). Quantification of sugar content was performed by calibration with external standards.

### 4.6. Statistical Analyses

All experiments were performed in three repetitions (except HPLC determinations of lactose hydrolysis products, performed with two repetitions). Statistical analyses of the composite synthesis models were performed using Design-Expert 13 (Stat-Ease, USA). Differences between means were analyzed with the Student’s *t*-test for independent samples with OriginPro 2022 were considered significant if the *p* value was below 0.05.

## 5. Conclusions

In the course of the presented work, the influence of PDMS as a modifying agent of silica composite on the activity of entrapped β-galactosidase was demonstrated. One of the major goals was to model and optimize the synthesis parameters to obtain a composite with a maximum activity of the entrapped enzyme. The dependencies were expressed with a reliable mathematical model, which facilitated the planning of the synthesis towards obtaining composites of desired final properties. These materials were characterized by their physicochemical properties and the activity of the entrapped enzyme. It was shown that PDMS improves the mechanical properties of the composite and increases the stability of β-galactosidase. Moreover, PDMS has been shown to influence the dominant mode of action of the enzyme.

Despite some the limitations of the described enzyme immobilization method (e.g., denaturation potential of silane precursors for some proteins, time required for aging and drying of the composite), the technique developed in this study opens the field for several new applications. It may be helpful when the immobilized protein shows hydrophobic properties that allow the interaction with PDMS, regardless of whether the protein has enzymatic properties or not.

## Figures and Tables

**Table 1:** Experimental matrix and results in the Box–Behnken design experiment. A—PDMS content (wt%); B—drying time (min); C—H_2_O:TMOS (molar ratio).

				Enzyme Activity (ALU/g)	
Run No.	A, PDMS Content (wt%)	B, Drying Time (min)	C, H_2_O:TMOS (Molar Ratio)	Actual	Predicted
1	0	90	30	15.48	15.47
2	20	30	10	6.54	6.51
3	40	90	10	5.79	5.80
4	0	30	20	12.83	13.26
5	20	150	30	18.49	18.52
6	40	30	20	18.41	18.43
7	20	90	20	22.00	21.85
8	20	150	10	2.13	2.55
9	20	90	20	21.24	21.85
10	40	90	30	15.87	16.28
11	0	150	20	16.64	16.63
12	20	90	20	21.73	21.85
13	20	90	20	22.14	21.85
14	20	90	20	22.16	21.85
15	20	30	30	14.45	14.03
16	40	150	20	16.01	15.58
17	0	90	10	2.88	2.47

**Table 2:** Analysis of variance (ANOVA) for the Box–Behnken design experiment—enzyme activity.

Source	SS	df	MS	*F*	*p*
**Model**	736.36	9	81.82	348.18	<0.0001
A-PDMS	8.51	1	8.51	36.23	0.0005
B-Drying	0.1340	1	0.1340	0.5704	0.4747
C-H_2_O:TMOS	275.72	1	275.72	1173.34	<0.0001
AB	9.68	1	9.68	41.19	0.0004
AC	1.59	1	1.59	6.76	0.0354
BC	17.83	1	17.83	75.89	<0.0001
A²	41.49	1	41.49	176.58	<0.0001
B²	31.60	1	31.60	134.50	<0.0001
C²	319.35	1	319.35	1359.00	<0.0001
**Residual**	1.64	7	0.2350		
Lack of Fit	1.05	3	0.3509	2.37	0.2115
Pure Error	0.5922	4	0.1480		
**Cor Total**	738.01	16			

**Table 3:** Summary of optimized parameters of β-galactosidase entrapping in silica composites.

Sample No.	PDMS Content [% *w*/*w*]	Drying time [min]	H_2_O:TMOS [mol·ratio]	β-gal. Content [mg/g]	Predicted Activity [ALU/g]	Observed Activity [ALU/g]
X1	22.9	90.8	20.1	1	22.01	21.98
X2	0.0	90.8	20.1	1	17.79	18.08
X3 (control)	22.9	90.8	20.1	0	n/a	n/a
X4 (control)	0.0	90.8	20.1	0	n/a	n/a

**Table 4:** Levels of variables and studied responses in the Box–Behnken design experiment.

Independent Variables	Levels			Response	
	–1	0	1		
A—PDMS content (wt%)	0	20	40	Gelation time [min]	Activity [ALU/g]
B—Drying time (min)	30	90	150		
C—H_2_O:TMOS (molar ratio)	10	20	30		

## Data Availability

Not applicable.
